# Isolation and characterization of stem cells from human exfoliated deciduous teeth

**DOI:** 10.6026/973206300200557

**Published:** 2024-05-31

**Authors:** Ratna Yumkham, C Nagarathna, Nelson Sanjenbam, Angom Gopilal Singh, Heisnam Philip Singh, Albert Ashem

**Affiliations:** 1 Department of Paediatric and Preventive Dentistry, Dental College, RIMS, Imphal, Manipur, India; 2 Department of Pedodontics and Preventive Dentistry, RajaRajeswari Dental College and Hospital, Bangalore, Karnataka, India; 3 Department of Oral and Maxillofacial Surgery Dental College, JNIMS, Porompat, Imphal, Manipur, India; 4 Braces Dental Care and 3D Imaging Centre, Imphal, Manipur, India; 5 Orthodontics and Dentofacial Orthopaedics Practioner, Imphal, Manipur, India; 6 Department of Oral Medicine and radiology, Dental College, RIMS, Imphal, Manipur, India

**Keywords:** Stem Cells, Multipotent Stem Cells, Dental pulp

## Abstract

SHEDs have been shown to have a higher rate of proliferation and raise in cell population doublings when compared to stem cells from permanent teeth. Hence, using them in tissue engineering may be advantageous over stem cells from adult human teeth. Stem cells were removed from pulpal tissues of thirty primary teeth undergoing extraction under six to fourteen year of age. The tissues were incubated after centrifuging and adding DMEM-KO following the addition of a 2 mg/ml collagenase blend for examination of plates in search of cell attachment and growth. Flow cytometric analysis showed successful isolation of SHEDs using fluoresce inisothiocyanate (FITC)-conjugated CD-34, CD-105, and PE (R-phycoerythrin)-conjugated CD-45, CD-90, CD-73, and HLA-DR antibodies. The surface antigens CD-73, CD-90 and CD-105 which are known to be present in mesenchymal lineages were positively expressed in SHEDs according to flow cytometry analysis, whereas CD-34, CD-45, and HLA-DR were not.

## Background:

Novel treatment approaches is required for best possible regeneration as well as repair of injuries and organs caused by illness, trauma, or birth defects. With the goal of repairing damaged, lost, aged, or malfunctioning cells as well as their extracellular matrices to restore tissue functions, the area of regenerative medicine seeks to address these demands. [1] Because adult stem cells don't involve the destruction of an embryo, as embryonic stem cells do, they eliminate ethical concerns. This has led to an increase in interest in adult stem cells in recent times. [2, 3, 4-5] Human exfoliated deciduous teeth (SHED) stem cells have recently been shown to represent a novel adult stem cell population with the capacity for multi-differentiation. In comparison to adult bone marrow MSCs as well as DPSCs (Dental Pulp Stem Cells), they were shown to have a greater proliferation rate as well as the ability to create numerous cell types in vitro, including odontogenic cells, neural cells, and adipocytes. [6] Therefore, it is of interest to describe the isolation and characterization of stem cells from human exfoliated deciduous teeth.

## Materials and Methods:

Thirty deciduous teeth that had been exfoliated and had a healthy pulp were obtained from children between the ages of six and fourteen. Informed consent was given by authorized representatives of each patient.

## Collection and transport of extracted teeth:

All the patients were asked to rinse their mouths with 0.2% chlorhexidine mouthwash. Extraction was carried out under standard conditions in local anesthesia. The extracted teeth had been cleaned with a sterile solution and brought to the tissue cultures lab in BD Falcon tubes filled with Dulbecco's Phosphate Buffer Saline (Invitrogen, USA).

## Isolation, digestion, and cultivation:

**Figure 1:**
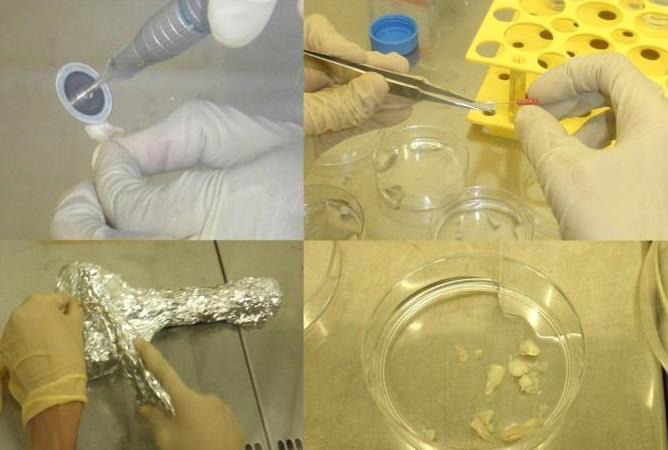
Methods of retrieving pulpal tissues

**Figure 2:**
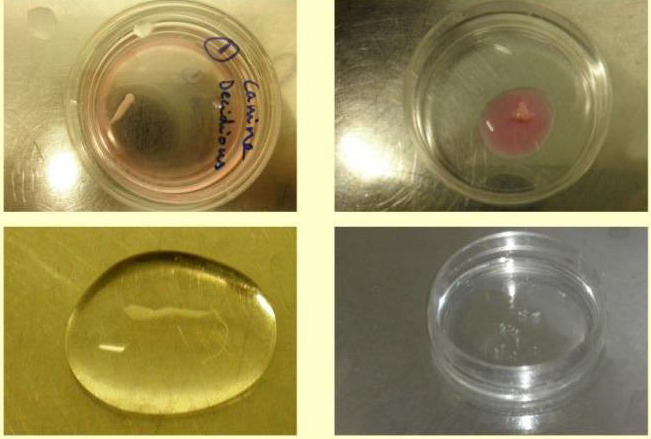
Retrieved dental pulp

These samples had been washed twice with PBS + 1% antimycotic inside the Biolaminar flow chamber. After that, the teeth were either broken into pieces with an osteotome wrapped in aluminum folds to make it easier to extract the pulpal tissues, or they had been placed inside a sterile surgical glove and access opened using a dental aerator (NSK) and a # 330 round diamond bur (Mani) (Figure 1). After that, the pulpal tissues had been extracted using tweezers or broaches and put in a 35 mm2 tissue culture flask (Figure 2). Following the addition of 2 mg/ml collagenase blend (Sigma) and tissue, the pulpal tissues had been extracted with tweezers or broaches and placed in 35 mm2 tissue culture flasks. The pulpal tissues had been then chopped using a surgical scalpel blade # 21 to maximize the enzyme's surface area of activity. For sixty minutes, the tissue was incubated at 37°C in a Heracel Thermo incubator. To reduce the enzyme's impact, DMEM-KO ("Dulbecco's Modified Eagles Medium-Knock out"), which contains "10% Foetal Bovine Serum (Hyclone), 100µM ascorbic acid, 2mM L-Glutamax, and supplements of 100U/ml penicillin and 100U/ml streptomycin, had been added" after incubation. After that, the samples were centrifuged for five minutes at 1800 rpm using an Eppendorf Centrifuge Machine 5415R from Germany. The supernatants had been disposed of, and the tissue pellets were plated in a 35 mm2 BD Falcon culture flask that was suitably labeled and contained 1 ml of DMEM-KO culture media. Finally, the cells had been incubated at 37°C in a humidified atmosphere with 95percent air and 5percent CO2. The incubator used was the Heracel Thermo. After 48 to 72 hours, the plates were examined again to look for cell attachment and development. Cell passaging and analysis of dental pulp cell surface molecules from exfoliated teeth were carried out in the laboratory using a panel of fluorochrome-labeled monoclonal antibodies that had been diluted in accordance with the manufacturer's instructions (Pharmingen).

CD 105, CD 90, and CD 73 are considered as positive markers. CD 34, CD 45, and HLA-DR were considered as negative markers.

 The isolated cells' surface phenotypic profile was ascertained by flow cytometric analysis. Detachable cells were counted. On the proper number of cells, 10µl of tagged primary antibody was added. As control groups, IgG2 (immunoglobulin G2) and IgG1 (immunoglobulin G1) isotopes were employed. On the ice, the cells were stained for one hour. Subsequently, 500µl of FACS buffer was pipetted thoroughly before being placed in tubes for flow cytometry. The flow cytometry machine was utilized to run the samples. BD The software CellQuestTM Pro Version 5.2.1 was utilized to examine the flow cytometric data. FITC-conjugated CD-34, CD-105, and PE (R-phycoerythrin)-conjugated CD-45, CD-90, CD-73, and HLA-DR antibodies had been utilized to stain the cells. For every marker, ten samples underwent the same procedure. The expression of cell surface marker expression from flow cytometric analysis was calculated as the arithmetic mean ± Standard Deviation (SD).

## Results:

## Isolation of Stem cells from SHED:

17 samples of human exfoliated deciduous teeth were effectively used to isolate stem cells. Following a cultivation period of 24 to 48 hours, single cells or tiny colonies of SHEDs were found. In primary culture, dental pulp cells had been seen to proliferate together with the formation of colonies. Fibroblastic cells made up the majority of the colonies. On top of the fibroblastic cells, several tiny, transparent cells were also visible. Ten days was the average time for the cultures to reach confluence. Confluent cultures, which are characteristic of MSC culture obtained from human bone marrow, consisted of several bundles of fibroblastic cells, each oriented in a certain direction.

## Flow cytometry analysis:

Flow cytometry analyses of SHED exhibited "high expression of the positive markers CD-73 [Graph 1], and CD-90 [Graph 2], and moderate expression had been seen for CD-105 [Graph 3], (Table 1). SHED progeny were negative for hematopoietic markers CD-34 [Graph 4], CD-45 [Graph 5], and HLA-DR" [Graph 6], (Table 2).

## Discussion:

MSCs can be isolated from different tissues and the advantages and disadvantages of these tissues are also there. [7- 8, 9, 10, 12] MSCs extracted from human deciduous teeth that were exfoliated have emerged as a compelling substitute in tissue engineering, according to Miura *et al.* (2003). [13] Since SHEDs have been shown to have a greater rate of proliferation and rise in "cell population doublings in comparison with stem cells from permanent teeth, using them in tissue engineering may be advantageous over using stem cells from adult human" teeth. [13] This could make it easier for these cells to proliferate in vitro prior to transplantation. Furthermore, in younger patients, SHED cells are extracted from a tissue that is easily accessible and "disposable," or regularly discarded. According to Nor JE (2006), it is advantageous to use dental pulp stem cells for young patients who are suffering from trauma-related pulp necrosis in their permanent incisors which are immature. [14] Because the patients' primary molars are at different stages of exfoliation due to their mixed dentition, SHED is a timely excellent source of stem cells for the engineering of dental pulp in immature permanent teeth.

The mesenchymal progenitors that were separated from the pulp of human deciduous incisors or SHED showed significant multi-potency since they were able to transform into osteoblasts, chondroblasts, and adipocytes. [13] SHEDs were successfully isolated and identified in the current study. Because barbed broach is more practical when used on single-rooted teeth, we could isolate SHEDs primarily from the main anterior teeth. Another likely explanation is that primary molars have bigger root bases, which allow them to remain in the mouth longer and resorb more slowly. This can lead to an obliterated pulp chamber that is devoid of pulp & stem cells. [15]

Dividing the tooth into pieces with an osteotome, which makes it easy to access the pulp tissues with a broach or Luer's forceps, is another method we've successfully utilized to remove pulp from the tooth. Conversely, we were unable to separate SHED from teeth that had been broken with a diamond disc. In those circumstances, we assume that there was extreme mechanical stress and overheating of the dental pulp. [16] The physical qualities, phenotypic traits, and biological behavior of stem cells are used to identify them. Characteristically, stem cells have a spindle form, a big central nucleus, and several cytoplasmic processes that typically protrude from the outside. [17] The MSC population must display "CD-73, CD-105, and CD-90 phenotypically, as determined by flow cytometry, as per the International Society for Cellular Therapy. Furthermore, CD-45, CD-34, CD-14 or CD-11b, CD-79a or CD-19, and HLA class II expression must be absent (5/2% positive) in these cells. Data shows CD-73, CD-90 and CD-105 are positive markers and CD-34, CD-45 and HLA-DR are negative markers. [18]

Data shows that phenotypical analysis of SHED revealed significant "positivity for the positive markers CD-73 (96.69%) and CD-90 (97.70%), and low positivity for CD-105 (34.33%), which is frequently expressed by endothelial" progenitors. This aligns with the findings of previous research. [16, 19] Because the cultured SHED cells did not display the negative markers HLA-DR (0.58%), CD-45 (0.88%), and CD-34 (1.76%), they are not hematopoietic. It was discovered that the phenotypic expression of CD-105 rose with a rise in passage number in one of the research done on dental pulp stem cells. By using passages p5 to p6, flow cytometry revealed that the expression was less than 40%, whereas using passages p8 to p10 resulted in an expression of more than 55%. [19] This could be a plausible reason for the mild phenotypic expression of CD-105 in the current investigation relative to other MSC markers, given that the flow cytometry was carried out during early passage. SHEDs were reported to express CD-105 well in a different investigation, which was not the case here. However, in that study, an additional incubation period was used with a secondary mAb (goat FITC-labeled against mouse Abs, Serotec). [20] Nevertheless, no such further incubation was done for this investigation. The differences in CD-105 expression could be explained by variations in the flow cytometry procedure. However, more research is needed to substantiate these claims. Furthermore, although being employed in immuno-magnetic selection for human MSCs, CD-105 is predominantly linked to endothelial cells. [21] We found multiple limitations during the isolation of SHEDs. One drawback was that in the pulp chamber of primary teeth that had been exfoliated, pulp tissue was not present. Pulp-derived cell cultures were not successful because of the small volume of pulp tissue. The other restriction was the contamination of cultures brought on by teeth-induced contamination. Contamination may arise after tooth extraction or during transport to the laboratory for cell culture. [16]

## Conclusion:

Dental pulp tissue of primary teeth which are exfoliated and normally discarded can be a readily available source of MSCscan and be used for many future studies and clinical uses. This provides fresh insights into the management of periodontal and pulp inflammation.

## Figures and Tables

**Figure 3:**
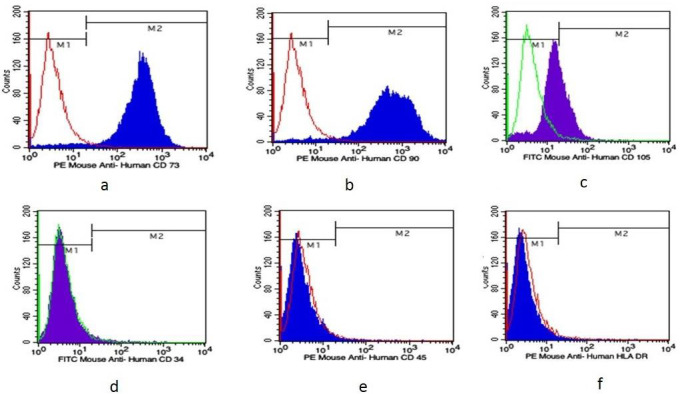
Immuno-phenotype analysis of SHEDs; (a) CD73; (b) CD90; (c) CD105; (d) CD34; (e) CD45 and HLA DR

**Table 1:** Positive Marker-Expressing Cells in Dental Pulp (In percentage)

**Cell markers**	**N**	**Mean**	**Standard Deviation**
CD 73	10	96.69	0.89
CD 90	10	97.7	0.72
CD 105	10	34.33	0.95

**Table 2:** Negative Marker-Expressing Cells in Dental Pulp (In percentage)

**Cell markers**	**N**	**Mean**	**Standard Deviation**
CD 34	10	1.76	0.11
CD 45	10	0.88	0.06
HLA-DR	10	0.58	0.05
